# Retention of Ciprofloxacin and Carbamazepine from Aqueous Solutions Using Chitosan-Based Cryostructured Composites

**DOI:** 10.3390/polym16050639

**Published:** 2024-02-27

**Authors:** Marinela-Victoria Dumitru, Ana-Lorena Neagu, Andreea Miron, Maria Inês Roque, Luisa Durães, Ana-Mihaela Gavrilă, Andrei Sarbu, Horia Iovu, Anita-Laura Chiriac, Tanța Verona Iordache

**Affiliations:** 1 National Institute for Research & Development in Chemistry and Petrochemistry-ICECHIM, 202 Spl. Independenței, 060021 Bucharest, Romania; marinela.dumitru@icechim.ro (M.-V.D.); ana-lorena.ciurlica@icechim.ro (A.-L.N.); andreea.miron@icechim.ro (A.M.); ana.gavrila@icechim.ro (A.-M.G.); andrei.sarbu@icechim.ro (A.S.); 2 Faculty of Chemical Engineering and Biotechnology, University POLITEHNICA of Bucharest, 1-7 Ghe. Polizu Street, 011061 Bucharest, Romania; horia.iovu@upb.ro; 3 University of Coimbra, CERES—Chemical Engineering and Renewable Resources for Sustainability, Department of Chemical Engineering, Rua Silvio Lima, 3030-790 Coimbra, Portugalluisa@eq.uc.pt (L.D.)

**Keywords:** cryostructured composites, chitosan, ciprofloxacin, carbamazepine, kinetics, water purification

## Abstract

Water pollution is becoming a great concern at the global level due to highly polluted effluents, which are charged year by year with increasing amounts of organic residues, dyes, pharmaceuticals and heavy metals. For some of these pollutants, the industrial treatment of wastewater is still relevant. Yet, in some cases, such as pharmaceuticals, specific treatment schemes are urgently required. Therefore, the present study describes the synthesis and evaluation of promising cryostructured composite adsorbents based on chitosan containing native minerals and two types of reinforcement materials (functionalized kaolin and synthetic silicate microparticles). The targeted pharmaceuticals refer to the ciprofloxacin (CIP) antibiotic and the carbamazepine (CBZ) drug, for which the current water treatment process seem to be less efficient, making them appear in exceedingly high concentrations, even in tap water. The study reveals first the progress made for improving the mechanical stability and resilience to water disintegration, as a function of pH, of chitosan-based cryostructures. Further on, a retention study shows that both pharmaceuticals are retained with high efficiency (up to 85.94% CIP and 86.38% CBZ) from diluted aqueous solutions.

## 1. Introduction

Numerous hazardous effluents, containing dyes, toxic heavy metals, inorganic anions, pesticides, cosmetics and pharmaceuticals are continuously polluting surface and ground water. In this context, industrial activities are known to be the main source of pollution [1], but recently, it was discovered that agriculture is also responsible for the discharge of large amounts of agrochemicals, organic matter, drug residues and pathogens [2]. In line with these two main pollution sources, the pharmaceutical sector contributes significantly to the pollution of water as there are still unsolved procedures for collecting and destroying the drugs that have passed their expiration date. Thus, many of them end up at municipal garbage sites, polluting the soil and surface water, or in the municipal wastewater flux, in which case the treatment procedures are not always adequate to clean the water from such pollutants [3]. In addition, large amounts of veterinary and human pharmaceuticals, and their metabolites, are found in the physiological remains of patients under treatment [4], which are again challenging for wastewater treatment plants.

In this respect, many researchers have expressed their concern about the regular discovery of antibiotics like ciprofloxacin (CIP) in surface and drinking waters all around the world [5], signalling that other treatment options should be implemented for cleaning up the antibiotic-contaminated waters [6,7,8]. CIP is a fluoroquinolone antibiotic also bearing a piperazine moiety. This antibiotic has been used since the 1980s and was reported to be one of the top selling antibiotics in 1990s (more than EUR 1 billion) [9]. CIP can be administrated to humans and in veterinary medicine for the treatment of a huge number of bacterial infections (like urinary [10], gastrointestinal [11], bone [12] and soft tissue infections [13], etc.), preventing their microbial activities. Several studies have confirmed that CIP represents 73% of the whole consumption of antibiotics in Europe [14,15].

On the other hand, some of the pharmaceuticals are persistent organic pollutants that cannot be eliminated by standard treatment facilities. One such example is the removal of carbamazepine (CBZ) through wastewater treatment facilities (WWTPs), which is typically less than 10% [16,17,18]. CBZ (or 5*H*-dibenzo[*b,f*]azepine-5-carboxamide) is an effective drug often used for controlling epileptic seizures [19] or in the treatment of epilepsy and psychotropic activity [20]. This type of drug has been used since the 1960s for severe pain, and after 1997, its consumption was almost 6334 kg in Austria alone [21]. In this context, water quality [22], ecosystems [23], and human health [24] are eventually impacted by drug residues in the environment [25]. For instance, it has been noted that CBZ is hazardous, even at doses lower than 100 mg/L, to aquatic organisms, animals and humans [26].

Therefore, it is critical to look for more effective solutions to remove pharmaceuticals, particularly CBZ and CIP, from wastewater [27,28,29]. For this reason, many studies in the literature have been focused on the development of new materials for CIP and CBZ retention and water purification [30,31,32,33,34]. Among those materials, alginate composite hydrogels [35], three-dimensional reduced graphene oxide/TiO_2_ aerogels [36], three-dimensional Co–N/SBA-15/alginate hydrogels [37] and sodium alginate gel beads [38] have been mentioned. However, most of the reported materials that were tested for their capacity to remove pharmaceuticals from wastewaters were quite efficient, especially for high concentrations of CIP and CBZ [39,40,41]. For instance, Zhao et al. [42] described the efficiency of a molecularly imprinted polyvinylidene difluoride membrane to retain CBZ in the 10–150 mg/L concentration range, revealing an experimental adsorption equilibrium of 4.359 mg CBZ/g of the adsorbent. Aghababaei et al. [43] used biobased adsorbents for retaining CBZ from solutions of 50 mg/L at 20 °C and achieved a higher adsorption capacity of 33 mg CBZ/g of the adsorbent. Other authors like Laishram Saya et al. [39] reported the use of a magnetic guar gum-grafted graphene oxide nanocomposite for CIP adsorption at a high concentration range of 100–700 mg/L. Nevertheless, the pollution issue remains, even when small concentrations of such pharmaceuticals are present in the water stream. In this respect, few publications present the efficiency of novel adsorbent materials for retaining CIP and CBZ at concentrations lower than 20 mg/L [44,45]. 

In recent years, a major part of the studies have presented different types of materials based on polymers with a gel structure [46]. Polymeric gels have a couple of crucial characteristics, including swelling, pseudoplastic (non-Newtonian) rheological behaviour, electrical oscillation, a mechanoelectrical effect, and interactions with opposingly charged surfactants [47,48,49,50]. As a result, various significant types of gels, including physical gels, hydrogels [51], nanogels [52], aerogels [53] and cryogels [54], have been studied, produced, and used in a variety of industrial applications over the past few years, including wastewater treatment [55,56]. However, for water treatment applications, the use of cryostructured materials instead of hydrogels may lead to higher adsorption capacities as a result of their extended pore structure, which facilitates access to specific binding sites. Furthermore, the procedures for designing cryostructures is less expensive than for preparing aerogels, in which case supercritical drying is used to extract the solvent [53].

Thereby, this study contributes to the state of the art with innovative cryostructured composites prepared from low-cost materials like chitosan (obtained from shrimp shell waste) and silicates (found in nature or prepared in the laboratory) used as potential adsorbent materials for removing persistent pharmaceuticals such as CIP and CBZ from aqueous solutions. The study presents the possibility to improve some of the mechanical features of the crystructured composites to withstand the harsh conditions in water treatment processes, followed by a proper investigation into such cryostructures to retain effectively the two targeted pharmaceuticals from diluted aqueous solutions. Additionally, the study also aims to prove that chitosan prepared in the laboratory containing native minerals is more efficient in preparing cryostrucured materials to retain various pharmaceuticals compared to commercial chitosan, which is more expensive as a result of the additional purification procedures (i.e., demineralization).

## 2. Materials and Methods

### 2.1. Materials

**Scheme 1:**
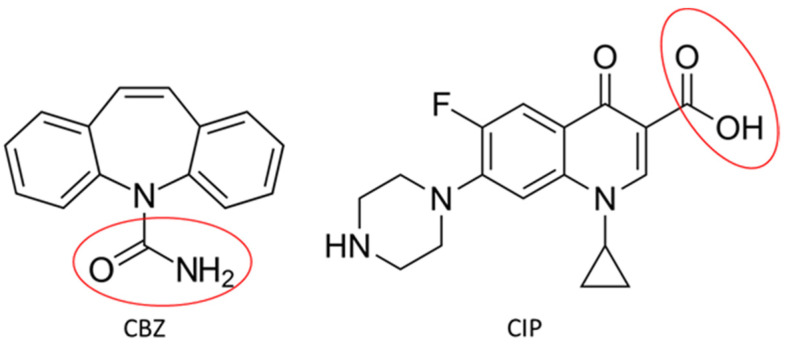
Structure of targeted pharmaceuticals, CIP antibiotic and CBZ drug, highlighting the active groups circled in red.

Two types of chitosan were used in this study, i.e., (i) commercial chitosan (C1, ≥75% deacetylation degree, Mn = 2.056 × 10^5^ g/mol, supplied by Sigma-Aldrich and used as received) and (ii) chitosan prepared from shrimp shells in a laboratory (C2, 76% deacetylation degree, Mn = 9.058 × 10^3^ g/mol). Kaolin (K, Acros Organics, Geel, Belgium) was previously silylated using a silane coupling agent γ-methacryloxypropyltrimethoxysilane (MAPTES, Sigma-Aldrich, 98% purity, St. Louis, MO, USA), while the organo-silicate (OS) microparticles were prepared via the sol–gel method, using tetraethyl orthosilicate (TEOS, 98%, Fluka, Fluka Chemie GmbH, Buchs, Switzerland), 3-aminopropyl triethoxysilane (APTES, 99%, Acros Organics, Pittsburgh, PA, USA), and sodium dodecyl-sulphate (95%, Scharlab, Barcelona, Spain), and ethanol (99.5%) and ammonium hydroxide (25%) supplied from ChimReactiv SRL-Bucharest, Romania. Standard solutions of pH 4, pH 7 and pH 9 (supplied by Metrohm Ltd., Herisau, Switzerland) were used to determine the swelling degrees. For the dissolution of chitosan, a mixture of glacial acetic acid (99%, Sigma-Aldrich) and distilled water was used. Ammonium bicarbonate (NH_4_HCO_3_, 99.5%, Sigma-Aldrich) was used as a foaming agent. Ciprofloxacin (CIP, purity ≥ 98%, M = 331.346 g/mol, Alfa Aesar, Haverhill, MA, USA) and carbamazepine (CBZ, M = 236.27 g/mol, Sigma-Aldrich), the structures of which are given in Scheme 1, are the two targeted pharmaceuticals for separation in wastewaters. 

### 2.2. Synthesis of Cryostructured Composites

The cryostructured composites were prepared in several stages, similar to the procedure described by Dumitru et al. [57], with the difference that the amount of the reinforcing material was kept constant at an optimum ratio of 1:2 (K-MAPTES/OS–chitosan). The silicates (e.g., K-MAPTES [57] and OS [58]) were added into the cryostructured matrix to improve their mechanical stability and resilience to disintegration in water at lower pH values. In the first step, the reinforcing materials K-MAPTES and OS were prepared. In this respect, K-MAPTES was synthesised using the instructions of Dumitru et al. [57], via the silanization of dried K with MAPTES (wt. ratio of 1:5) at 110 °C for 24 h, while the silicate microparticles (noted as OS) were prepared according to the procedure depicted in the study by Neagu et al. [58]; in the latter, a typical sol–gel reaction with basic catalysis using TEOS and APTES (mol. ratio of 1:1) was applied, and a microemulsion was formed and stabilized by the addition of sodium dodecyl sulphate. In a subsequent step, 0.3 g of chitosan (C1 or C2) was dissolved in 12 mL of acetic acid solution (98% H_2_O—2% CH_3_COOH for C1, 90% H_2_O—10% CH_3_COOH for C2) followed by K-MAPTES or OS additions (50% wt. relative to chitosan). The foaming agent was added in the weight ratio of 1:2 chitosan/NH_4_HCO_3_ by vigorous mechanical stirring. The obtained foams were immediately frozen (at −20 °C for 24 h) and lyophilized (at −50 °C for 48 h) to obtain the two targeted cryostructured composite series with K-MAPTES (noted as C1-K and C2-K) and with OS (noted as C1-OS and C2-OS). Some representative pictures of foams and cryostructured composites are given in Figure S1 (Supplemental Material).

### 2.3. Characterization Techniques

Fourier Transform Infrared Spectrometry (FTIR)

The FTIR spectra were recorded using a ThermoScientific Summit Pro (Waltham, MA, USA) spectrophotometer, performing 16 scans for each sample at a resolution of 4 cm^−1^, in the spectral range of 4000–400 cm^−1^. The samples were analysed and diluted in potassium bromide pellets.

Scanning electron microscopy (SEM)

SEM images were recorded using a Quanta Inspect F scanning electron microscope (Waltham, MA, USA) equipped with an emission gun and a 1.2 resolution field (EGF). Aiming at confirming the success of silicate incorporation within the chitosan matrix, SEM images were recorded for all the cryostructured composite series. The samples were placed on a carbon strip and further attached to a copper grid. The samples were coated for 30 s with a thin layer of gold using the sputter coater Q150R ES Plus (Quorum). The gold coating was achieved uniformly by obtaining an electrically conductive thin film (~film thickness 5 nm), thus inhibiting “charging”, reducing thermal damage, and increasing the emission of secondary electrons.

Mechanical tests

The mechanical tests were performed with the Inspekt mini-series equipment (Hegewald & Peschke, Nossen, Germany), performing uniaxial compressions with a strain rate of 1 mm min^−1^, using a 50 N cell. The mechanical properties of the samples, the stiffness (Young’s modulus) and destructiveness (compression to the limit test), were evaluated from the loading stage.

Determination of Swelling Degrees (SDs)

The swelling degrees (*SDs*) of cryostructured composites were determined until disintegration, which depended on the pH value of the aqueous solution. This behaviour was studied to help explain the properties of the cryostructured adsorbents in the following retention trials for CIP and CBZ. Therefore, the cryostructured composite samples were soaked in 10 mL of standard pH solutions (4, 7 and 9) in Falcon tubes with a capacity of 50 mL. The stirring was maintained by the MultiTherm shaker device (Cool-Heat-Shake, Benchmark Scientific, Sayreville, NJ, USA) Benchmark (200 rpm, 22 °C). The *SDs* were calculated at different time intervals according to Equation (1), where *m*_s_ (g) and *m*_d_ (g) represent the weight of the swollen and dried adsorbents, respectively.

(1) $$SD=\frac{m_{s}-m_{d}}{m_{d}}$$

Retention capacity of cryostructured composites for CBZ and CIP

The prepared cryostructured adsorbents were tested for their capacity to retain CIP and CBZ using synthetic aqueous solutions. For this trial, the UV–Vis spectra were recorded using a T70+ UV–Vis spectrophotometer. Both solutions of pharmaceuticals were ultrasonicated at 25 °C for 1 h while ensuring light protection. For this procedure, a Bandelin Sonorex Digiplus type DL 102 H device with a capacity of 3 L was used at an 80% sonication power (28 kHz). Each adsorbent (approximately 0.01 g) was contacted with a volume of 10 mL of CBZ (10 mg/L) or CIP (6 mg/L) solution. At different time intervals (5, 10, 20, 60, 120, 240 and 1440 min), the supernatant was tested via UV–Vis spectroscopy at *λ* = 284 nm (specific wavelength of CBZ) and *λ* = 273 nm (specific wavelength of CIP) in order to determine the adsorption kinetics. The calibration equations for CIP and CBZ quantifications are given in Figure S2 (Supplemental Material). For the cryostructured series with calcium carbonate-enriched chitosan, the quantification of CIP was performed assuming that no calcium CIP salt was formed and retained in the matrix during the adsorption assays. The retention capacity of the cryostructured composites for CBZ and CIP (*q*, mg (CBZ/CIP)/g adsorbent) was calculated using Equation (2), while the yield of retention (*Y*, % (CBZ/CIP)) was determined using Equation (3). The kinetics of the adsorption was fitted using a pseudo-first-order model [59], a pseudo-second-order model [60], the intraparticle diffusion model [61] and the Elovich linear model [62] according to Equations (4)–(7), respectively.

(2) $$q\left(\frac{mg}{g}\right)=\left(C_{i}-C_{f}\right)\;V_{s}/m_{adsorbent}$$

(3) $$Y\left(\%\right)=\frac{\left(C_{i}-C_{f}\right)}{C_{i}}\times 100$$

where *C*_i_ (mg/L) and *C*_f_ (mg/L) are the initial and final concentrations of CBZ and CIP in the supernatant, *V*_s_ (L) represents the volume of the (CBZ and CIP) solution, and *m*_adsorbent_ (g) represents the weight of the dried adsorbent.

(4) $$q_{t}=q_{e}(1-e^{{-k}_{1}t})$$

where *q*_e_ is the amount of pharmaceutical adsorbed at equilibrium (mg/g), *q*_t_ is the amount of pharmaceutical adsorbed at time *t* (mg/g), and *k*_1_ is the pseudo-first-order rate constant (min^−1^).

(5) $$\frac{t}{q_{t}}=\frac{t}{q_{e}}+\frac{1}{k_{2}q_{e}^{2}}$$

where *q*_e_ and *q*_t_ represent the adsorption capacity (mg/g) at equilibrium and at time *t* (min), respectively, and *k*_2_ (g/mg/min) is the pseudo-second-order adsorption rate constant [57].

(6) $$q_{t}=k_{p}t^{1/2}+C$$

where *k*_p_ is the intraparticle diffusion rate constant (mg/g/min^1/2^), *q*_t_ is the amount of pharmaceutical adsorbed at time *t* (mg/g), and *C* is the constant related to the thickness of the boundary layer (mg/g).

(7) $$q_{t}=\frac{1}{\alpha }\ln\left(\alpha \right.\left.\beta \right)+\frac{1}{\alpha }\ln t$$

where *q*_t_ is the amount adsorbed of pharmaceutical at time *t* (mg/g), *α-* is the initial adsorbent rate (mg/g/min), and *β*- is the desorption constant during each experiment (mg/g).

## 3. Results and Discussion

### 3.1. Synthesis of Cryostructured Composites

Similar to the previous work [57], this study emphasises the superior properties of cryostructured composites prepared with a type of mineral-enriched chitosan from shrimp shell waste (C2) [63], as will be further presented. The synthesis protocol of this type of chitosan by-passes the demineralization step of chitin, which is only deproteinated and deacetylated to deliver mineral-enriched chitosan, as described in the study of Miron et al. [63]. The results obtained for one type of mineral-enriched chitosan adsorbent system [57], which refers to improved stability in water up to 4 h (at pH 5.5) and a high penicillin G retention up to 24 h (at pH 6.5), have encouraged further investigations in terms of retaining other types of pharmaceuticals and varying the nature of the reinforcement materials. Referring to the improvement of the mechanical stability and water resistance, cryostructured composite series with commercial chitosan were prepared and compared to the target cryostructured chitosan. For the same reason, a similar system composed of chitosan and modified K, as described in reference [57], was synthesised and tested for CIP and CBZ retention. Advancing the work undertaken previously, in an original attempt to increase the stability of cryostructured composites in water and to evaluate the capacity of the thus-prepared adsorbents for retaining other types of pharmaceuticals, a set of cryostructured composites was additionally prepared using organo-silicate (OS) microparticles instead of K-MAPTES. The rationale behind this change in the reinforcement material is related to the hypothesis that smaller particles such as OS (200–400 nm) can be better dispersed in the cryostructure matrix and thus can improve the bulk properties of the composite in terms of mechanical stability and water resistance at lower pH values. Additionally, it can also be mentioned that both types of reinforcement materials were functionalized with organic moieties (i.e., methacryloxy groups in MAPTES or aminopropyl groups in APTES), able to interact with hydroxyl groups in chitosan, for ensuring a higher compatibility with the matrix. The following interpretation of the adsorption behaviour presumed that the functional organic moieties of silicates do not participate in the adsorption process of CIP and CBZ. 

### 3.2. Structure and Morphology of Cryostructured Composites

**Figure 1:**
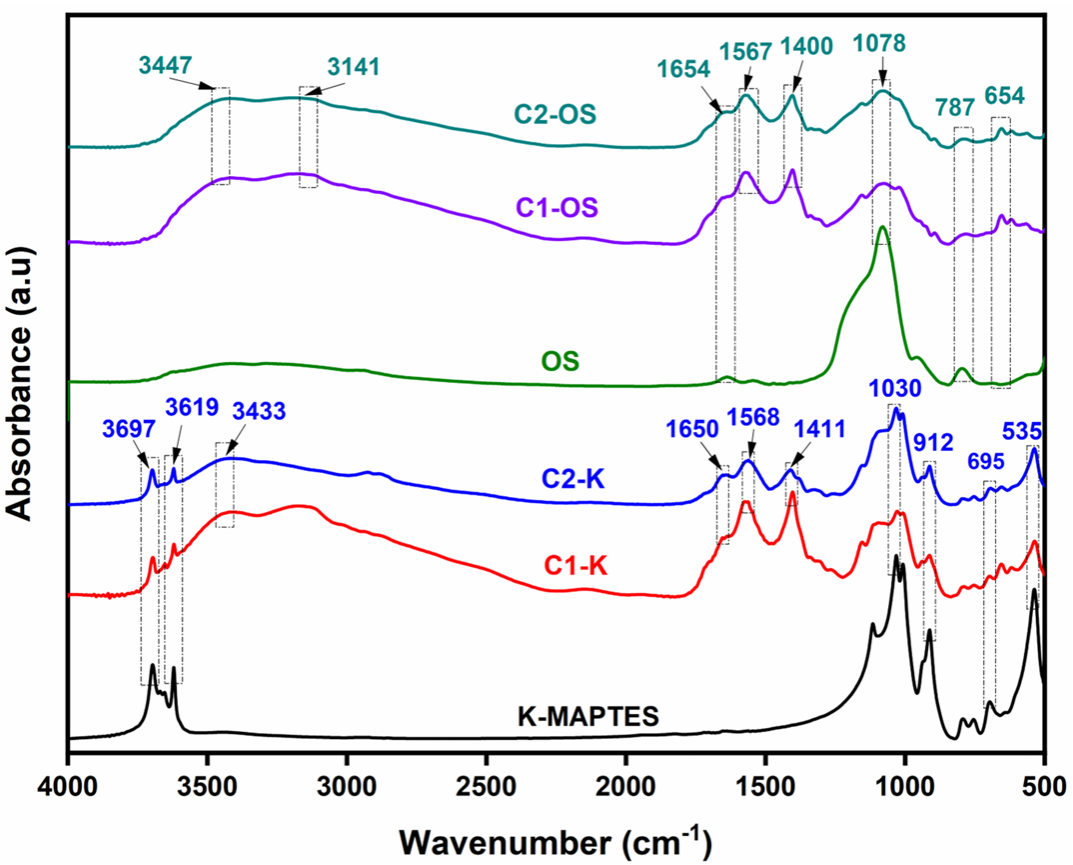
FTIR spectra for cryostructured composite series C1-K and C2-K compared to K-MAPTES and series of C1-OS and C2-OS compared to OS, respectively.

The FTIR spectra for the composite materials prepared using the two types of chitosan (C1 and C2) and two different silicates (K-MAPTES and OS) are given in Figure 1, while the characteristic bands were summarized for convenience in Table S1 (Supplemental Material). The cryostructured composite with OS or K-MAPTES content presents characteristic bands for O-H stretching vibrations (at 3697 cm^−1^ and 3447 cm^−1^), N-H and O-H stretching vibration (at 3141 and 3433 cm^−1^), C=O stretching (at 1650 cm^−1^ and 1654 cm^−1^), N-H bending (at 1567 cm^−1^ and 1568 cm^−1^) and CH_2_ bending (at 1400 cm^−1^ and 1411 cm^−1^) attributed to the chitosan structure [64]. The amide band is more intense for cryostructures containing chitosan C1 compared with C2, thus confirming that C1 has a lower deacetylation degree. As it can be seen in Figure 1, K-MAPTES was successfully incorporated into the chitosan structure (C1-K and C2-K) due to the appearance of the characteristic band of Si-O-Si (around 1030 cm^−1^) and Si-O-Al (at 912 cm^−1^ and 695 cm^−1^) [65,66]. The same structure resemblance was also noticed in the series of cryostructures containing OS (C1-OS and C2-OS). For the OS structure, the bands corresponding to the stretching vibration of Si-O-Si can be observed at 1078 cm^−1^ and 787 cm^−1^. Therefore, the FTIR spectra contributed to understanding the composition for both types of chitosan-based cryostructures and to a confirmation of the incorporation of silicates into the matrix. 

**Figure 2:**
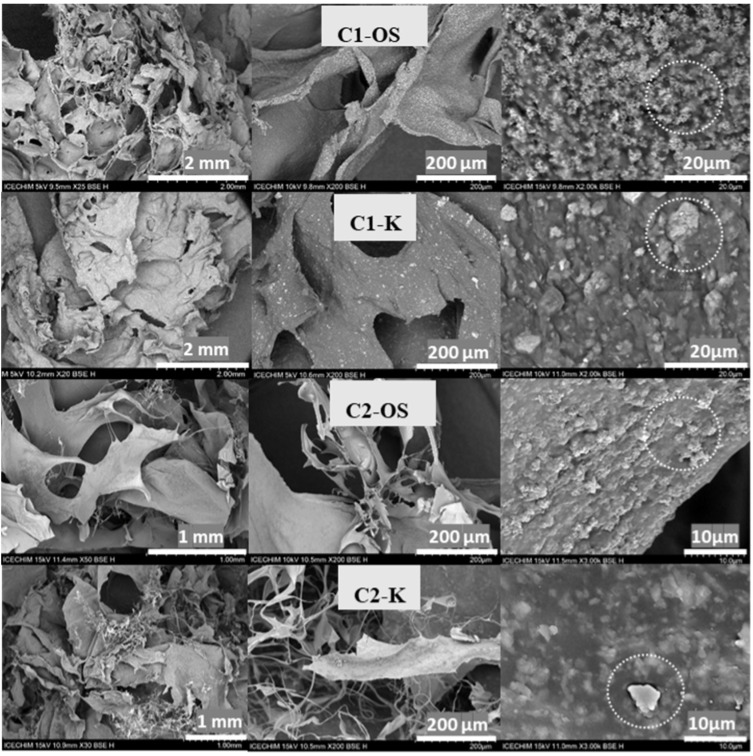
SEM images for cryostructured composite series based on commercial chitosan (C1-OS and C1-K) and chitosan prepared from shrimp shells (C2-OS and C2-K), highlighting the inclusion of K or OS (white circles).

Additional to FTIR, scanning electron microscopy (Figure 2) was used to underline the incorporation of silicates into the chitosan matrix and to visualize the macroporous structure of prepared cryostructures. The composites seem to present subtle differences as a result of the chitosan type but also due to the silicate used. The cryostructures prepared with commercial chitosan (C1) seem to be more compact with smaller pores and relative smooth inner surfaces, while the cryostructures with C2 present a more fibrillated morphology, as exposed at a 200 μm scale. Taking a closer look at the 10 and 20 μm scales, the presence of K-MAPTES, with a particle size of 1–2 μm, and that of OS microparticles, with particle diameters of around 200–400 nm, may be observed. As initially presumed, OS seem to be more homogenously dispersed into the chitosan matrix, while for K-MAPTES, a high degree of agglomeration was noticed, which may affect the overall mechanical stability and water resistance.

### 3.3. Mechanical Stiffness of Cryostructured Composites

All the samples were tested under a compressive load between different strain intervals, as it can be observed in Figure S3 (Supplemental Material). The samples became thinner and compressed (process of densification) due to the force applied (50 N cell) on their macroporous structure but were not destroyed completely after being analysed. From the collected data, it can be concluded that C1-K did not require much strength for structure failure, unlike OS-based cryostructures and C2-K. The maximum strain for samples C2-K, C2-OS and C1-K was higher (between 84 and 122%) in comparison with C1-K, for which a lower strain of 5.5% was recorded when fracture occurred. The results suggest again that chitosan from shrimp shells is one factor that contributes to the improvement of mechanical features, together with the fact that OS leads to higher homogeneity and hence improved bulk mechanical stability, regardless of the chitosan type. Young’s moduli present the following values, taken via the slope of the initial linear part of the curve (elastic region): C1-K—13 kPa; C1-OS—40 kPa; C2-K—33 kPa; and C2-OS—167 kPa. It can be seen that the lowest value was recorded for sample C1-K, which can be associated with the almost instant failure of the structure. Following this, the other three samples were far more rigid, with improved values of Young’s moduli, compared to bare chitosan (7 MPa) and the composites thereof, as depicted in other studies [67]. The compression stress–strain behaviour of the samples closely relates with the findings reported in the study by Buchtova et al. [68], which conducted a compression analysis on cryogels prepared from cellulose using a similar approach. Notably, both sets of cryostructures were prepared using natural polymers, which confirmed that cryostructures derived from natural polymers exhibit a shared compression response characterized by a similar stress–strain curve. However, the results from this current study indicate a strain range of 85–120% for the cryostructures based on chitosan, somewhat higher than the findings in [68], where the maximum strain was approximately 85%.

### 3.4. Evaluation of the Swelling Degrees (SDs) at Various pH Values

**Figure 3:**
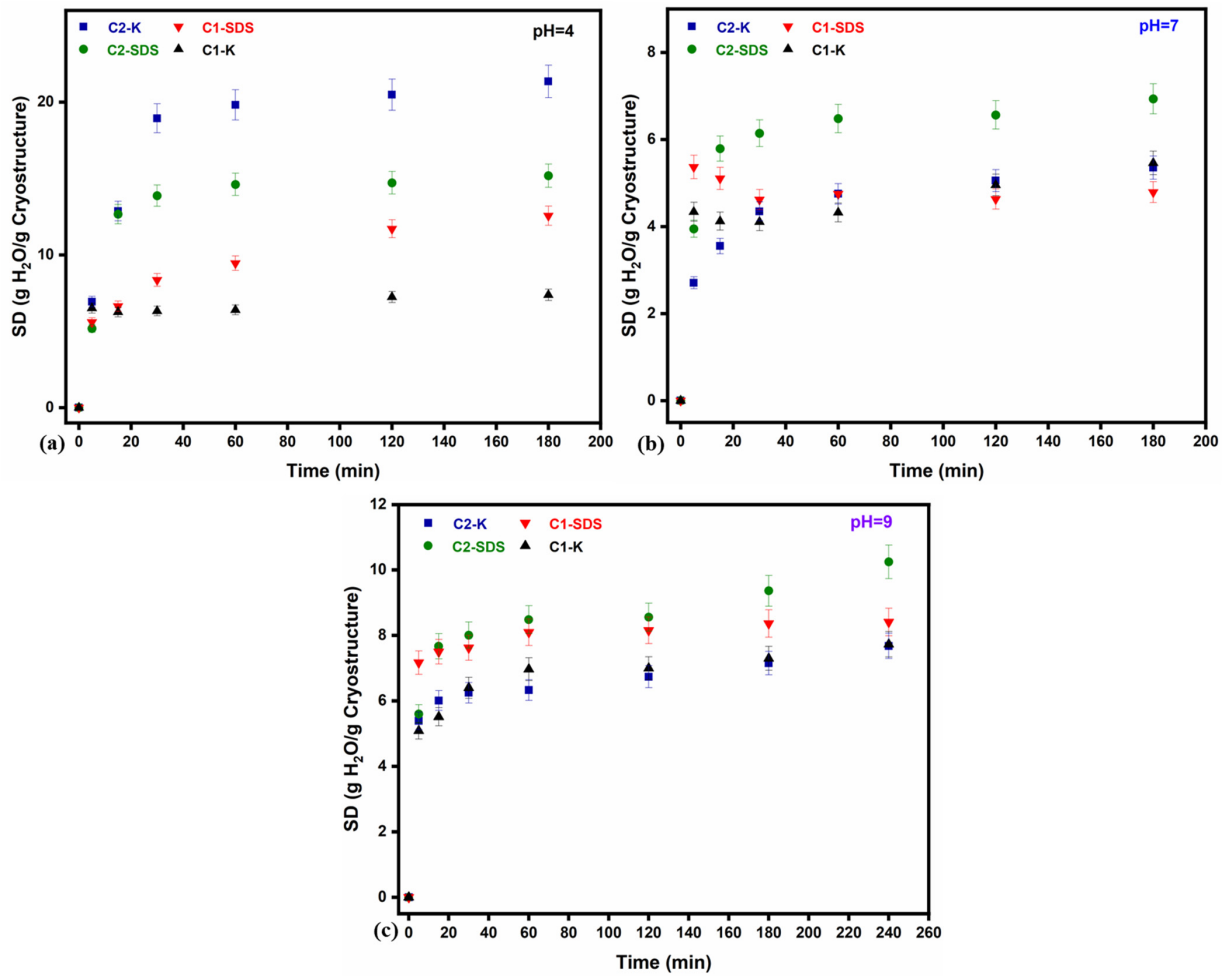
Variation in *SDs* as a result of time (5% error bars of measured data) for the cryostructured composites series based on C1 and C2, performed in standard solutions at different pH values ((**a**) 4, (**b**) 7, (**c**) 9) and room temperature.

Because former studies [57] have shown that such cryostructured systems are prone to disintegrate after only 1 or 2 h at pH 5.5, the swelling degrees (*SDs*) for all the composite cryostructures were evaluated at different pH values (4, 7 and 9), as presented in Figure 3. The variation in *SDs* in time confirmed the observations from former experiments, regarding the capacity of cryostructured composites to withstand disintegration for longer periods of time in aqueous solutions at higher pH values (from 2–3 h to 4 h or more). Again, the results point out that the composites based on chitosan prepared from shrimp shells (C2) were more resilient and registered, overall, higher swelling degrees compared to the commercial chitosan-based systems (C1-K and C1-OS). As expected, C2-OS registered lower water adsorption capacities compared to C2-K, due to its confined and stiffer structure. Overall, these properties show the superior mechanical resistance of C2-OS. 

The only disadvantage to the pH increase is related to the lower capacity of cryostructures to adsorb water, which, in this case, it was drastically reduced from 21.35 g water/g adsorbent, at pH 4, to 7.15 g water/g adsorbent, at pH 9, for C2-K and from 15.18 g water/g adsorbent to 9.36 g water/g adsorbent for CS-OS, respectively (as also summarized in Table S2, Supplemental Material). It can also be noticed that in between, at pH 7, all the systems registered an even lower performance compared to the results obtained at pH 4, where their resistance in water was expected to be higher (for at least 4 h). Therefore, to compensate for the negative effect of the pH on the swelling properties, the following retention studies were performed at pH 6.0 ± 0.5; this value of the pH seems to be optimum for other pharmaceuticals retention, as proposed by former studies for CIP and CBZ retention [44,45]. 

### 3.5. Evaluation of CBZ and CIP Retention via Batch Adsorption Measurements

**Figure 4:**
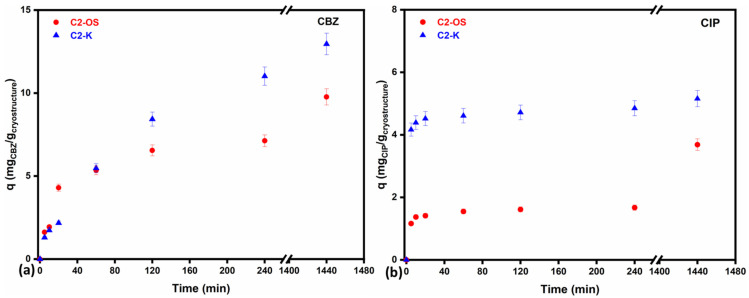
Variation in adsorption capacity across time (5% error bars of measured data) for cryostructured composites C2-K and C2-OS towards (**a**) CBZ and (**b**) CIP from aqueous solution of 15 mg/L for CBZ and 6 mg/L for CIP, respectively.

**Figure 5:**
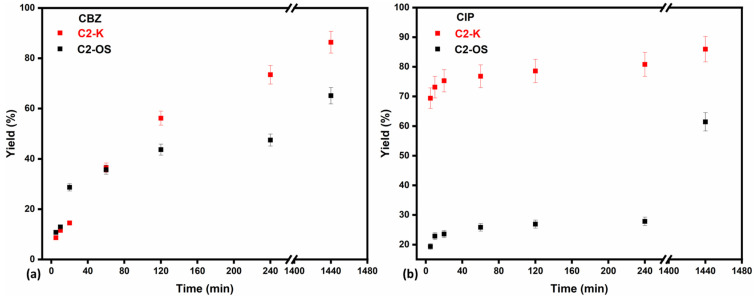
Yield of removal capacity across time (5% error bars of measured data) for cryostructured composites C2-K and C2-OS towards (**a**) CBZ and (**b**) CIP.

Since there was clear evidence that systems with commercial chitosan (C1) were not very stable, in this section, only the kinetic process for the two series representatives, C2-K and C2-OS, is evaluated and compared. In this respect, several experiments were conducted to study the effect of the contact time on the retention performance of composites for both targeted pharmaceuticals, CIP and CBZ, at pH 6.0 ± 0.5. As may be observed in Figure 4 and Table S3 (Supplemental Material), both cryostructured composites were able to withstand disintegration in the aqueous solution up to 24 h, which confirmed the proper selection of the working pH. Another worthy result refers to the maximum adsorption capacity after 24 h of C2-K for CBZ and CIP, up to 12.95 mg/g and 5.16 mg/g, respectively, compared to C2-OS, which registered maxima of 9.77 mg/g and 3.69 mg/g of the same pharmaceuticals. This difference is in fact explainable and correlates well with the results obtained for the *SD* evaluation. The same properties that endow C2-OS with mechanical stability and water resistance limit the retention of the two pharmaceuticals, as also observed for water retention. In Figure 5, the yield of retention, *Y* (%), as a function of time for the two cryostructured composites (CBZ in Figure 5a and CIP in Figure 5b) reveals that both systems can retain important amounts of CIP or CBZ from the initial solution. As also summarized in Table S3 (Supplemental Material), maximum yields were registered after 24 h for C2-K, up to 86.38% CBZ and 85.94% CIP, in comparison to C2-OS, which managed to retain 65.11% CBZ and 61.63% CIP. 

Further on, the adsorption behaviour for CBZ and CIP was described by the pseudo-first-order, pseudo-second-order, intraparticle diffusion and Elovich kinetic models, given by Equations (4)–(7), respectively. The graphs were collected in Figures S4, S6, S8 and S10 for CBZ and Figures S5, S7, S9 and S11 for CIP in the Supplemental Material, while the parameters are summarized in Table 1. For the adsorption of CBZ, the kinetic model suitability was as follows: (i) pseudo-second-order model > pseudo-first-order model > Elovich > intraparticle diffusion model for the C2-K system and (ii) pseudo-second-order model > Elovich > pseudo-first-order model > intraparticle diffusion model for the C2-OS system. Whereas, for CIP adsorption, the obtained sequence was as follows: (i) pseudo-second-order model > pseudo-first-order model = Elovich > intraparticle diffusion model for the C2-K system and (ii) pseudo-second-order model > intraparticle diffusion model > Elovich > pseudo-first-order model for the C2-OS system. From this sequence, it can be noticed that the C2-K system led to similar adsorption mechanisms for both types of pharmaceuticals. Meanwhile, the C2-OS system presented quite different adsorption behaviour for CBZ and CIP when fitted to the different kinetic models.

The intraparticle diffusion model can be applied for porous materials, such as the ones obtained in this study, where the equilibrium is reached in the pores of the material via diffusion mechanisms [69]. This model describes quite well the kinetics of CIP in the C2-OS system. However, comparing the coefficients of determination, R^2^, in Table 1, it seems that more processes influence the adsorption equilibrium, such as chemisorption, which is why the Elovich kinetic model was also fitted to the obtained data. This model is suitable for systems with heterogeneous adsorbing surfaces, especially to prove that chemical adsorption (in terms of chemisorption) took place [41]. In this case, it may be observed that the Elovich model is more suitable for the CBZ adsorption but still does not describe the adsorption behaviour very well, especially for CIP (C2-OS). Although it is clear that the adsorption mechanism was not completely governed by a physisorption mechanism, the study also provides the data obtained for fitting the pseudo-first-order kinetic model. The pseudo-first-order model involves diffusion processes, in which case the rate of adsorption is influenced by the initial concentration of the adsorbate for single adsorbate systems [70]. As it can be observed from comparing the coefficients of determination, R^2^, in Table 1, the overall adsorption rate of pharmaceuticals is not only influenced by diffusion, especially regarding CIP adsorption (C2-OS). Therefore, to demonstrate that chemisorption may be the rate-limiting factor in all cases, the pseudo-second-order kinetic model was also plotted, which seem to be the most suitable model for the sorption mechanism for both types of pharmaceuticals by C2-K and C2-OS, as well. Thus, the results indicate that the adsorption rate is rather influenced by the adsorption capacity instead of the initial concentration of the adsorbate [70] and that the two pharmaceuticals were adsorbed on the surface and within the pores of the composite materials. Previous studies in the literature also demonstrated similar trends in pharmaceuticals adsorption, with the best regressions achieved using the pseudo-second-order kinetic model for CIP [71] and CBZ [72,73].

As a final remark, changing the reinforcement material K-MAPTES using a synthetic silicate (OS) led to some clear improvements in the mechanical stability of physically crosslinked chitosan-based cryostructures but with an obvious loss of retention capacity for pharmaceuticals. Nevertheless, the results in both cases are promising compared to other studies, especially because the prepared cryostructured composites are efficient in removing CBZ or CIP from diluted aqueous solutions (15 mg/L CBZ and 6 mg/L CIP). In a recent study conducted by Al Ghoul et al. [44], activated carbon was used to retain CBZ from aqueous solutions in the range of 2.5–20 mg/L, for which an equilibrium adsorption capacity of 2.46 mg/g was obtained, while in the present study, both types of cryostructured materials attained higher adsorption capacities for CBZ, up to 12.95 mg/g and 9.77 mg/g for C2-K and C2-OS, respectively. Another recent study has also highlighted that smectite–chitosan nanocomposites were able to retain 50.8 mg CIP/g adsorbent, with a similar removal yield (86.7%) as obtained in this present study (85.94%) when using higher initial concentrations of 10 mg CIP/L [45].

## 4. Conclusions

This study was focused on studying the effect of the reinforcement material upon the stability and removal properties of chitosan-based cryostructured composites. To this aim, two types of chitosan and two types of silicates were combined to develop cryostructures for CIP antibiotic and CBZ drug retention from diluted aqueous solutions. Changing the functionalized kaolin particles (K-MAPTES) with sol–gel-derived microparticles (OS) led to an improvement in both mechanical and water resistance of the cryostructured composites, due to the more homogenous structure that formed upon this addition. In this respect, FTIR and SEM micrographs provided information about the incorporation of the silicates in the chitosan matrix, which ultimately led to some changes in the surface morphology and porosity of the thus-prepared cryostructured composites. Linked to the improved stability of cryostructures, chitosan prepared in the lab from shrimp shells, by-passing the demineralization step (C2), also contributed to a more compact and resistant structure, which performed well under compressive stress. Further on, the swelling study at three different pH values revealed that the optimum working pH that favours the adsorption of water and the targeted pharmaceuticals is somewhere between pH 4 and pH 7. Therefore, performing the retention trials at pH 6.0 ± 0.5 led to some progress in terms of adsorption capacity, comparable to other studies. The C2-K cryostructures were able to adsorb high quantities of pharmaceuticals, up to 12.96 mg CBZ/g and 5.15 mg CIP/g, with yields of 86.38% CBZ and 85.94% CIP. As determined by the regression models, the best fitted kinetic model for the cryostructured composite systems was the pseudo-second-order model, which highlighted a major contribution of chemisorption upon the sorption mechanism of both pharmaceuticals. Thus, the proposed adsorbent cryostructured composites can become potential candidates as adsorbent materials for CIP and CBZ retention in water purification procedures.

## Figures and Tables

**Table 1:** Parameters for the four investigated kinetic models for CBZ and CIP.

Pharmaceutical Code	CBZ			CIP		
	Pseudo-first-order kinetic model (Equation (4))					
Sample Code	*K* (g mg^−1^ min^−1^)	*q*_e_ (mg g^−1^)	*R* ^2^	*K* (min^−1^)	*q*_e_ (mg g^−1^)	*R* ^2^
C2-K	8.50 × 10^−3^	12.95	0.992	430.00 × 10^−3^	5.15	0.980
C2-OS	18.00 × 10^−3^	9.76	0.927	68.00 × 10^−3^	3.68	0.490
	Pseudo-second-order kinetic model (Equation (5))					
Sample Code	*K*_2_ (g mg^−1^ min^−1^)	*q*_e_ (mg g^−1^)	*R* ^2^	*K*_2_ (g mg^−1^ min^−1^)	*q*_e_ (mg g^−1^)	*R* ^2^
C2-K	9.30 × 10^−4^	12.95	0.995	17.17	5.15	0.999
C2-OS	1.98 × 10^−3^	9.76	0.998	36.35	3.68	0.984
	Intraparticle diffusion kinetic model (Equation (6))					
Sample Code	*K*_p_ (mg g^−1^ min^−1/2^)	*C* (mg g^−1^)	*R* ^2^	*K*_p_ (mg g^−1^ min^−1/2^)	*C* (mg g^−1^)	*R* ^2^
C2-K	0.33	2.16	0.752	0.02	4.34	0.833
C2-OS	0.21	2.73	0.789	0.07	0.96	0.959
	Elovich linear kinetic model (Equation (7))					
Sample Code	*α* (mg g^−1^ min^−1^)	*β* (mg g^−1^)	*R* ^2^	*α* (mg g^−1^ min^−1^)	*β* (mg g^−1^)	*R* ^2^
**C2-K**	0.42	−3.42	0.949	6.16	3.96	0.980
**C2-OS**	0.68	−0.74	0.979	2.72	0.28	0.703

## Data Availability

The original contributions presented in the study are included in the article and the Supplementary Material, further inquiries about the raw data can be directed to the corresponding authors.

## Supplementary Materials

The following supporting information can be downloaded at: https://www.mdpi.com/article/10.3390/polym16050639/s1, Figure S1: A typical foaming process of (a) chitosan–silicate mixture and (b) frozen cryostructured sample; Figure S2: Calibration curves for (a) ciprofloxacin and (b) carbamazepine; Table S1: FTIR spectral assignment of bands for each cryostructured composite series; Figure S3: Compression tests results for cryostructured composites based on C1 (C1-K, C1-OS) and on C2 (C2-K, C2-OS); Table S2: Swelling degrees of the two cryostructured composite series performed at different values of pH (4, 7 and 9); Table S3: Adsorption capacities, *q* (mg/g adsorbent), and removal yield, *Y* (%), of cryostructures for CBZ from 15 mg/L solution and for CIP from 6 mg/L solution in batch mode; Figure S4: Non-linear regression for the pseudo-first-order kinetic model towards CBZ; Figure S5: Non-linear regression for the pseudo-first-order kinetic model towards CIP; Figure S6: Linear regression for the pseudo-second order kinetic model towards CBZ; Figure S7: Linear regression for the pseudo-second-order kinetic model towards CIP; Figure S8: Linear regression for the intraparticle diffusion kinetic model towards CBZ; Figure S9: Linear regression for the intraparticle diffusion kinetic model towards CIP; Figure S10: Linear regression of Elovich kinetic model for CIP data adsorption; and Figure S11: Linear regression of Elovich kinetic model for CBZ data adsorption.
