# Supplementary material for: Retention of Ciprofloxacin and Carbamazepine from Aqueous Solutions Using Chitosan-Based Cryostructured Composites
Source: Polymers (Basel). 2024 Feb 27;16(5):639. doi: 10.3390/polym16050639 (PMC10934589; doi:10.3390/polym16050639)
Supplement: Supplementary file 1 [file polymers-16-00639-s001.zip › polymers-2851998-supplementary.pdf]

polymers

Supplemental Material

Retention of Ciprofloxacin and Carbamazepine from Aqueous Solutions Using Chitosan-Based Cryostructured Composites

Marinela Victoria Dumitru [1,2], Neagu Ana-Lorena [1], Andreea Miron [1], Maria Inês Roque [3], Luisa Durães [3], Ana-Mihaela Gavrilă [1], Andrei Sarbu [1], Horia Iovu [2], Anita-Laura Chiriac [1,*] and Tanța Verona Iordache [1,*]

[1] National Institute for Research & Development in Chemistry and Petrochemistry-ICECHIM, 202 Spl. Independenței, 060021 Bucharest, Romania; marinela.dumitru@icechim.ro (M.V.D.); ana-lorena.ciurlica@icechim.ro (N.A.-L.); andreea.miron@icechim.ro (A.M.); ana.gavrila@icechim.ro (A.-M.G.); andrei.sarbu@icechim.ro (A.S.)
[2] Faculty of Chemical Engineering and Biotechnology, University POLITEHNICA of Bucharest, 1-7 Ghe. Polizu Street, 011061 Bucharest, Romania; horia.iovu@upb.ro
[3] CIEPQPF, Department of Chemical Engineering, University of Coimbra, R. Silvio Lima, 3030-790 Coimbra, Portugal; luisa@eq.uc.pt (L.D.)
* Correspondence: anita-laura.radu@icechim.ro (A.-L.C.); tanta-verona.iordache@icechim.ro (T.V.I.)

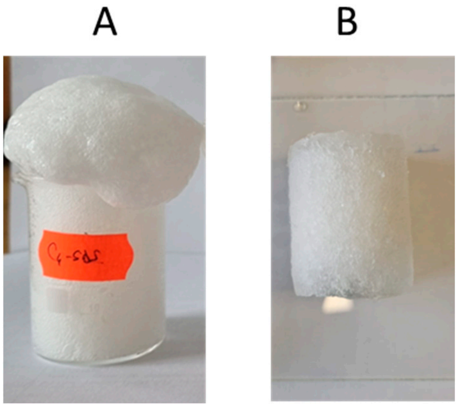

Figure S1. A typical foaming process of (**A**) chitosan-silicate mixture and (**B**) frozen cryostructured composite.

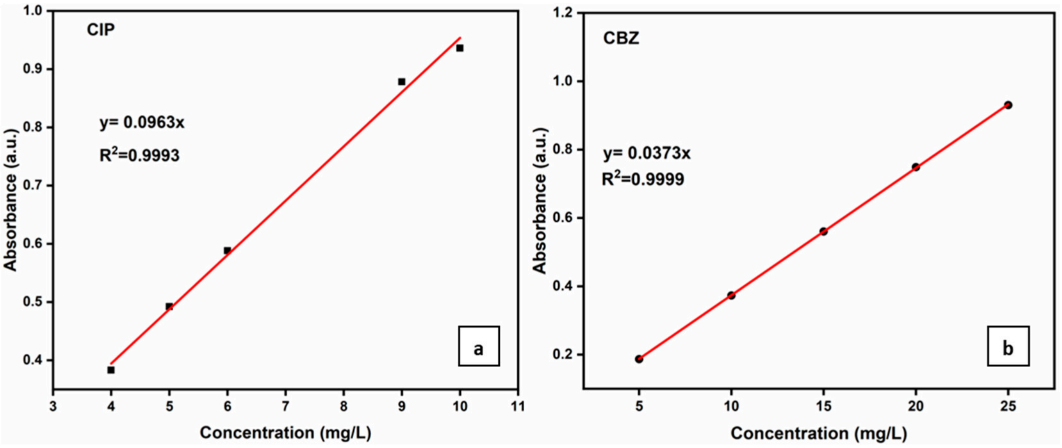

Figure S2. Calibration curves for (**a**) Ciprofloxacin and (**b**) Carbamazepine.

Table S1. FTIR spectral assignment of bands for each cryostructured composite series.

Sample Series	Wavenumbers (cm^{-1})	Spectral Assignments
**C1-K** **C2-K**	3697–3619	O-H from hydroxyl group (moisture)
	3433	O-H and N-H stretching vibration (chitosan structure)
	1650	C=O stretching of amide I from chitosan structure
	1568	N-H bending of amide II
	1411	CH_2 bending
	1030	Si-O-Si stretching from K-MAPTES structure
	912	Si-O-Al (Kaolin)
	695–535	Si-O-Si
**C1-OS** **C2-OS**	3447	O-H from hydroxyl group (moisture)
	3141	N-H stretching vibration (chitosan structure)
	1654	C=O stretching of amide I from chitosan structure
	1567	N-H bending of amide II
	1400	CH_2 bending
	1078	Si-O-Si stretching band from OS structure
	787–654	Si-O-Si from OS structure

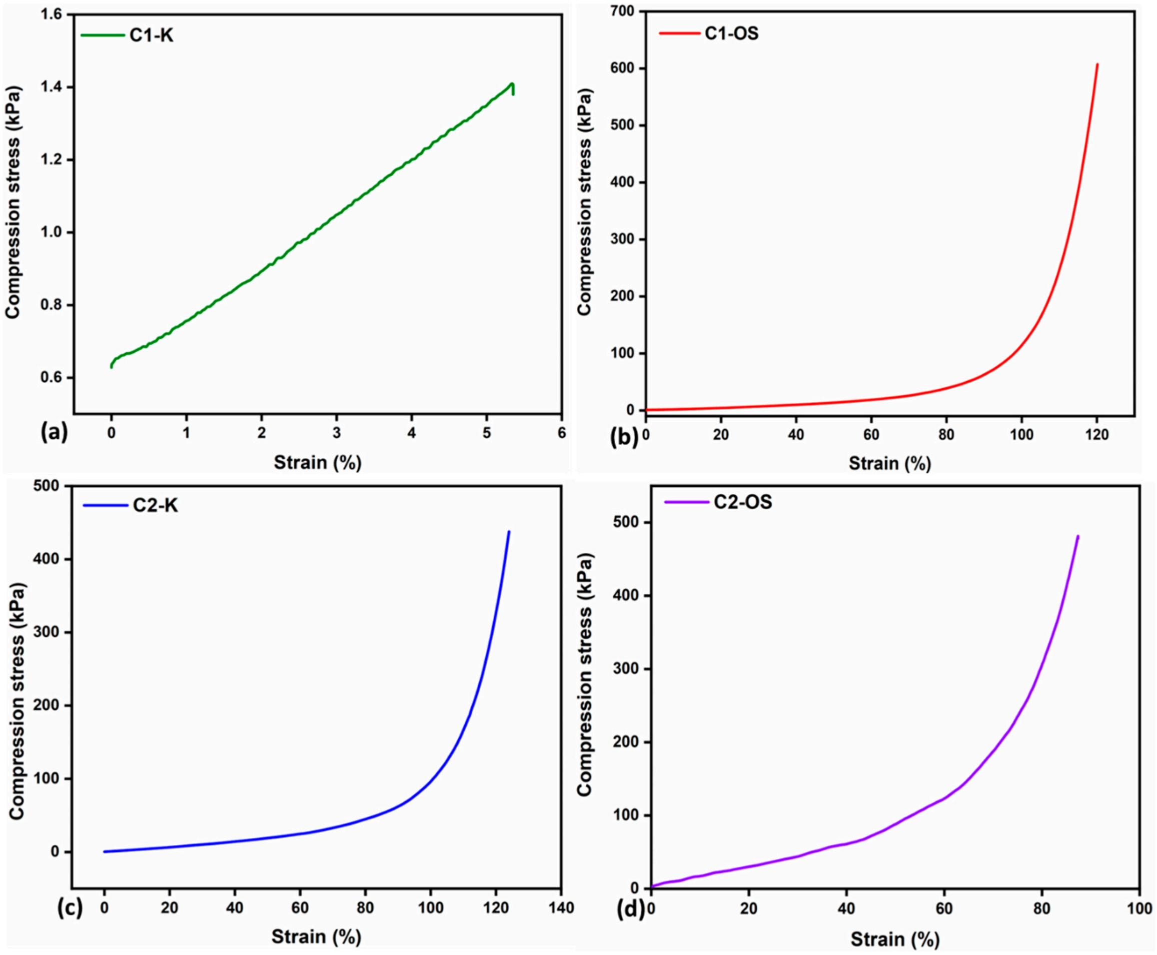

Figure S3. Compression tests results for cryostructured composites based on C1 (C1-K, C1-OS) and on C2 (C2-K, C2-OS).

Table S2. Swelling degrees of the two cryostructured composites series performed at different values of pH (4, 7 and 9).

Time (min)	*SD* (g H_2O/ g adsorbent) pH=4				*SD* (g H_2O/ g adsorbent) pH=7				*SD* (g H_2O/ g adsorbent) pH=9			
	C1-K	C2-K	C1-OS	C2-OS	C1-K	C2-K	C1-OS	C2-OS	C1-K	C2-K	C1-OS	C2-OS
5	6.51	6.93	5.60	5.18	4.34	2.71	5.37	3.95	5.09	5.39	7.17	5.60
15	6.25	12.86	6.64	12.66	4.12	3.55	5.10	5.79	5.51	6.01	7.50	7.67
30	6.32	18.93	8.35	13.87	4.11	4.35	4.62	6.14	6.40	6.25	7.62	8.01
60	6.39	19.81	9.45	14.61	4.32	4.75	4.75	6.48	6.97	6.33	8.10	8.48
120	7.24	20.47	11.70	14.72	4.95	5.05	4.63	6.56	7.00	6.73	8.15	8.56
180	7.38	21.35	12.55	15.18	5.46	5.35	4.79	6.93	7.30	7.15	8.36	9.36
240	-	-	-	-	-	-	-	-	7.73	7.68	8.40	10.24

Table S3. Adsorption capacities, *q* (mg/g $_{cryostructures}$), and removal yield, *Y* (%), of cryostructures for CBZ 15 mg/L solution, and for CIP 6 mg/L solution, in batch mode.

Time (min)	CBZ				CIP			
	q (mg/g adsorbent)		*Y* (%)		*q* (mg/g adsorbent)		*Y* (%)	
	C2-K	C2-OS	C2-K	C2-OS	C2-K	C2-OS	C2-K	C2-OS
5	1.30	1.62	8.66	10.79	4.14	1.16	69.40	19.36
15	1.73	1.94	11.56	12.93	4.39	1.31	73.13	22.85
30	2.18	4.30	14.52	28.67	4.52	1.41	75.29	23.55
60	5.48	5.35	36.51	35.65	4.61	1.55	76.82	25.82
120	8.42	6.55	56.17	43.66	4.71	1.61	78.58	26.89
240	11.01	7.12	73.43	47.49	4.85	1.67	80.80	27.84
1440	12.95	9.77	86.36	65.11	5.16	3.69	85.94	61.43

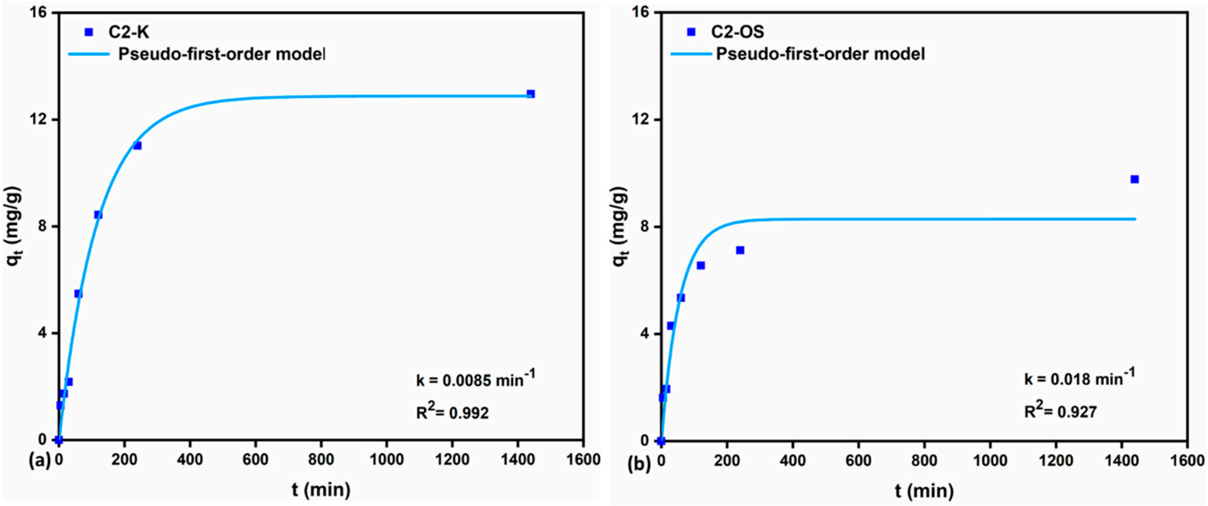

Figure S4. Non-linear regression for the pseudo-first-order kinetic model towards CBZ.

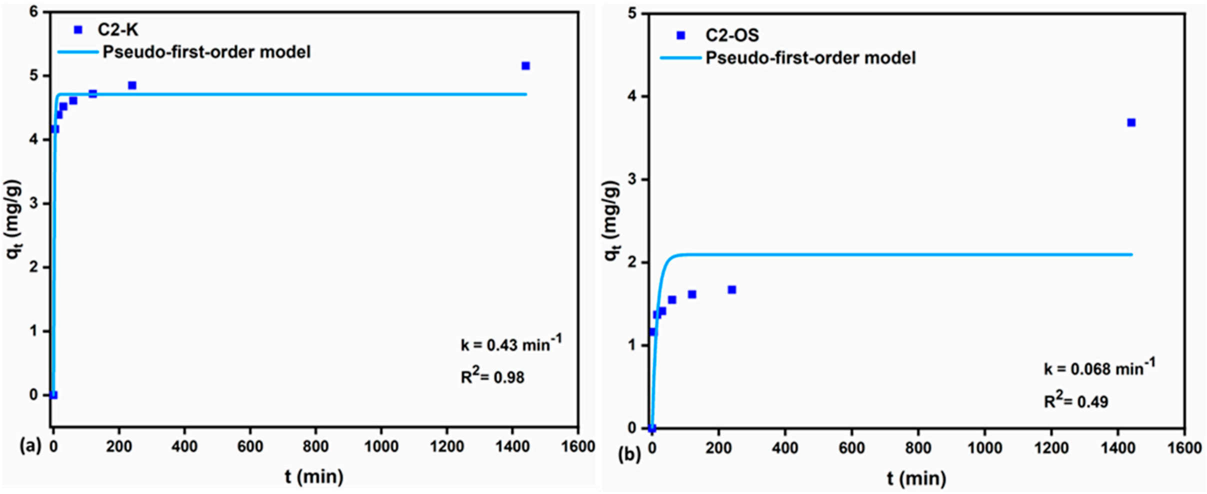

Figure S5. Non-linear regression for the pseudo-first-order kinetic model towards CIP.

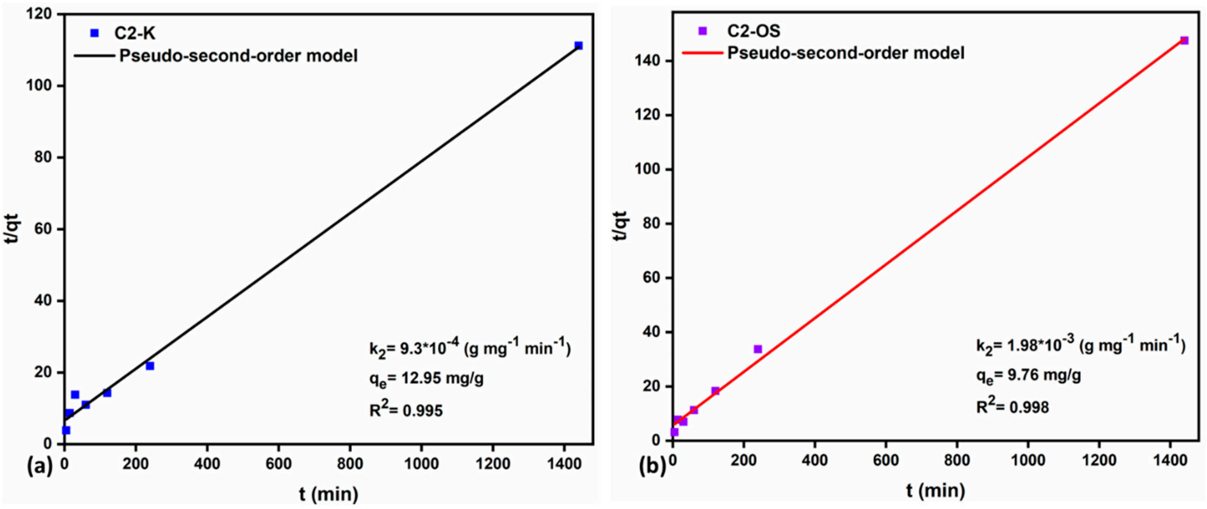

Figure S6. Linear regression for the pseudo-second order kinetic model towards CBZ.

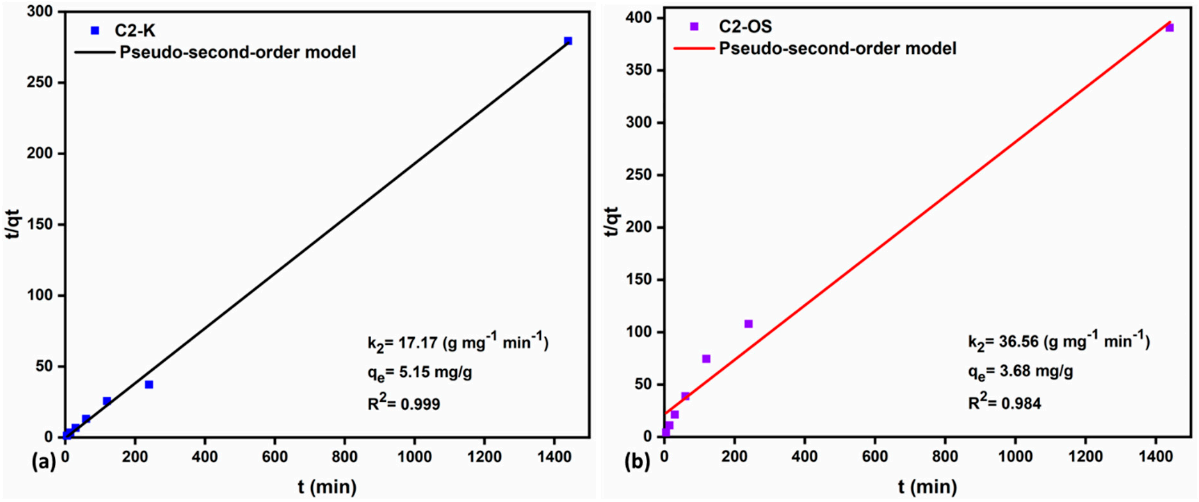

Figure S7. Linear regression for the pseudo-second-order kinetic model towards CIP.

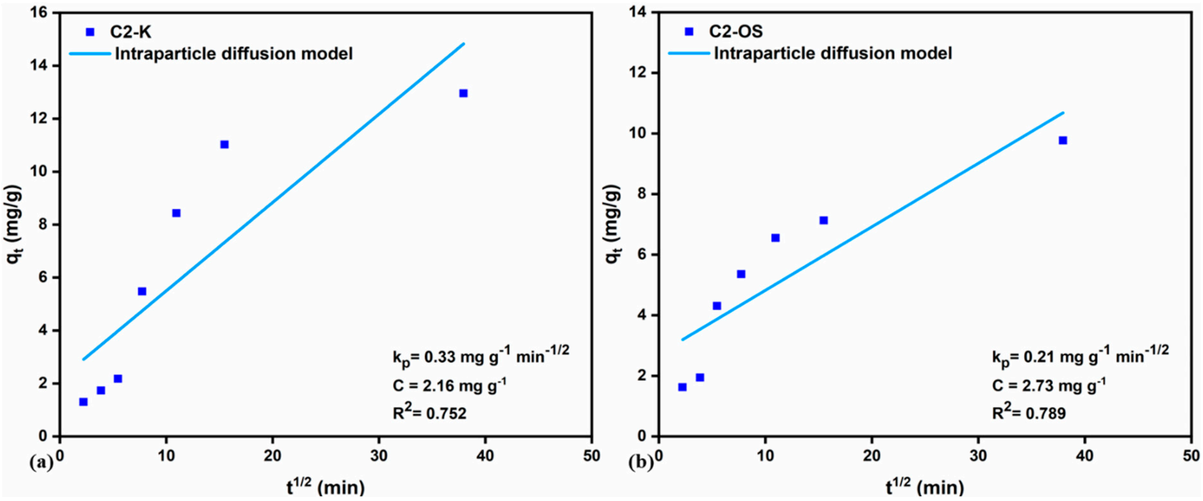

Figure S8. Linear regression for the intraparticle diffusion kinetic model towards CBZ.

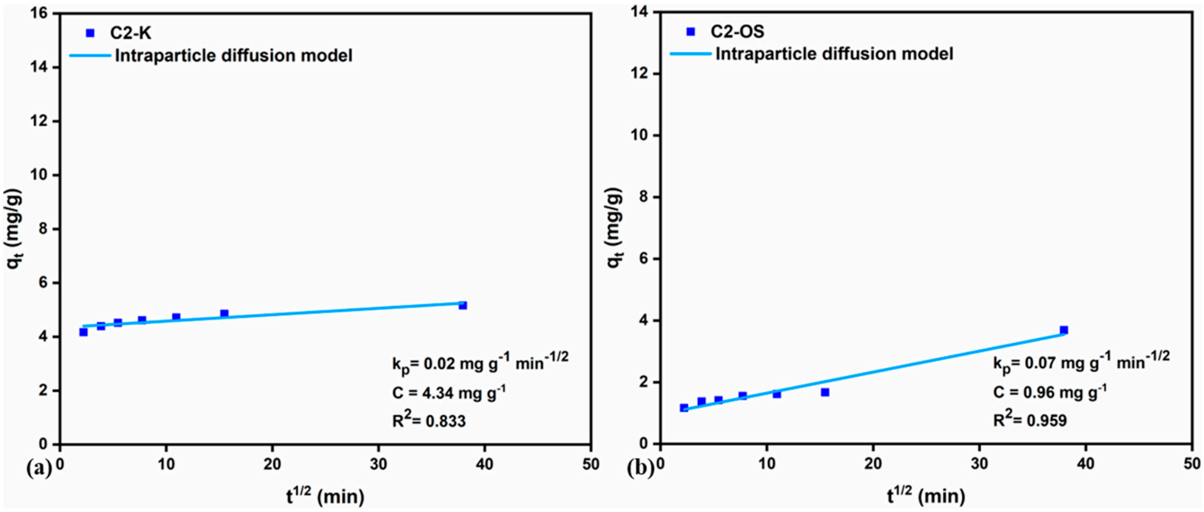

Figure S9. Linear regression for the intraparticle diffusion kinetic model towards CIP.

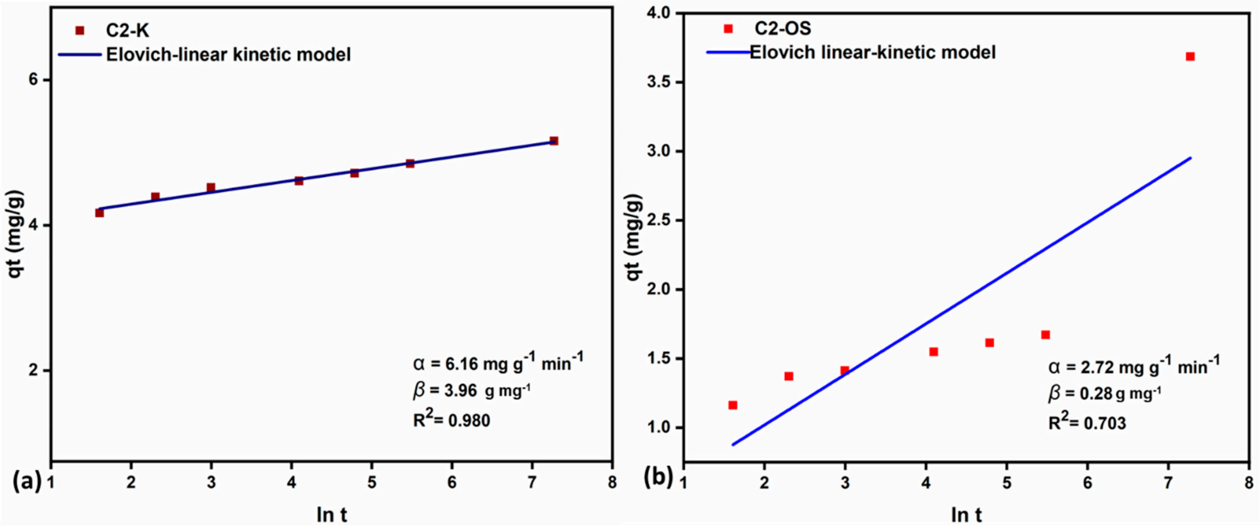

Figure S10. Linear regression of Elovich kinetic model for CIP data adsorption.

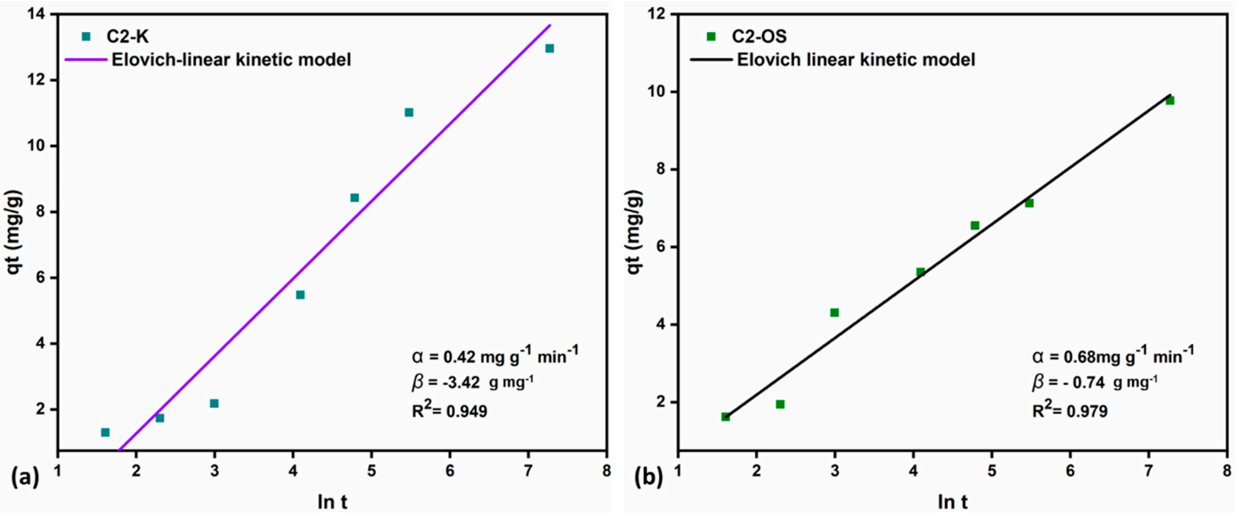

Figure S11. Linear regression of Elovich kinetic model for CBZ data adsorption.
